# Acute caval thrombosis leading to obstructive shock in the early post insertion period of an inferior vena cava filter: a case report and literature review

**DOI:** 10.1186/s12959-023-00567-3

**Published:** 2024-01-10

**Authors:** ShuTing Gao, YunFei Chen, YaTing Huang, YiPing Dang, YiQing Li

**Affiliations:** Department of Vascular Surgery, Union Hospital, Tongji Medical College, Huazhong University of Science and Technology, 1277 Jiefang Avenue, Wuhan, 430022 China

**Keywords:** Inferior vena cava thrombosis, Obstructive shock, Inferior vena cava filter, Pulmonary embolism, Anticoagulation, Orthopaedic trauma

## Abstract

**Background:**

Obstructive shock is extremely rare in clinical practice and is caused by acute blood flow obstruction in the central vessels of either the systemic or pulmonary circulation. Utilizing inferior vena cava filters (IVCFs) to prevent pulmonary embolism (PE) is associated with some potential complications, such as inferior vena cava thrombosis (IVCT). Shock as a direct result of IVCT is rare. We present a case of obstructive shock secondary to extensive IVCT caused by inadequate anticoagulant therapy after the placement of an IVCF.

**Case presentation:**

A 63-year-old male patient with a traffic accident injury presented orthopaedic trauma and lower limb deep vein thrombosis (DVT). He experienced sudden and severe abdominal pain with hypotension, tachycardia, tachypnea, oliguria and peripheral oedema 5 days after IVCF placement and 3 days after cessation of anticoagulant therapy. Considering that empirical anti-shock treatment lasted for a while and the curative effect was poor, we finally recognized the affected vessels and focused on the reason for obstructive shock through imaging findings—inferior vena cava thrombosis and occlusion. The shock state immediately resolved after thrombus aspiration. The same type of shock occurred again 6 days later during transfer from the ICU to general wards and the same treatment was administered. The patient recovered smoothly in the later stage, and the postoperative follow-up at 1, 3, and 12 months showed good results.

**Conclusion:**

This case alerts clinicians that it is crucial to ensure adequate anticoagulation therapy after IVCF placement, and when a patient presents with symptoms such as hypotension, tachycardia, and lower limb and scrotal oedema postoperatively, immediate consideration should be given to the possibility of obstructive shock, and prompt intervention should be based on the underlying cause.

## Introduction

In clinical practice, the appropriate use of an IVCF has proven effective in intercepting lower limb DVT emboli, thereby preventing the occurrence of PE. However, as the utilization of IVCFs has become more common and follow-up durations have extended, an increase in the incidence of associated complications has also been observed. Current research reveals that complications arising from filter placement encompass inferior vena cava (IVC) narrowing and occlusion, perforation of the IVC, filter tilt, fracture, and migration. Stenosis and occlusion of the IVC primarily result from thrombosis. Based on the degree of obstruction, most cases of IVCT formation exhibit no prominent acute symptoms, although a minority might present with severe acute manifestations. Instances of obstructive shock caused by IVCT are still rare. Obstructive shock entails acute circulatory obstruction within the central vasculature of either the systemic or pulmonary circulation. Not only is the incidence of obstructive shock rare, but there is also a paucity of literature regarding this condition.

This paper reports the case of a patient with lower limb DVT who developed IVCT following IVCF insertion and subsequently suffered obstructive shock at our centre. The paper also provides a succinct review of the relevant literature.

## Case presentations

**Fig. 1:**
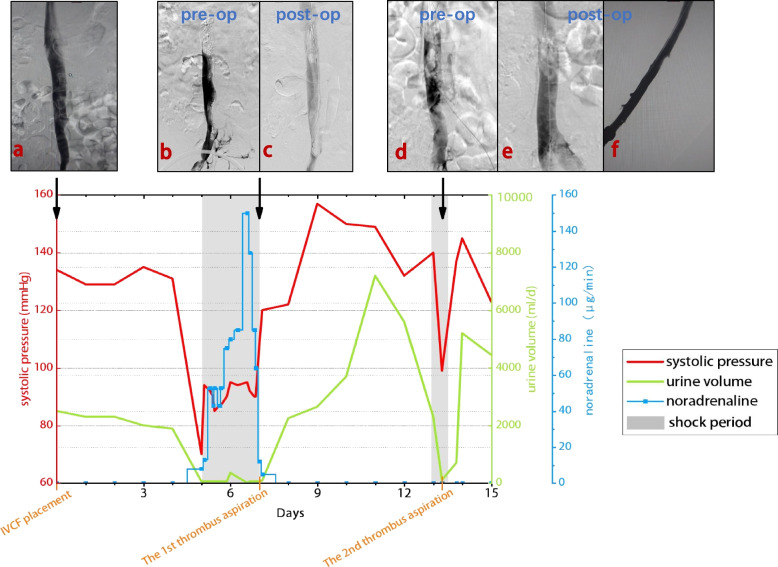
The line graph depicts the variations in blood pressure, urine output, and norepinephrine usage during the patient’s course of illness. Following the first thrombus aspiration procedure, upon partial recanalization of the IVC, blood pressure and urine output rapidly normalized, accompanied by a sharp decrease in the requirement for vasopressor medications. Subsequent to the second aspiration procedure, similar immediate improvements were observed in blood pressure and urine output. Intraoperative angiographic imaging following DENALI™ filter placement, illustrating unobstructed IVC return flow(**a**). Intraoperative angiographic images from the first thrombus aspiration procedure (b and c): The pre-aspiration angiography revealed obstruction within IVC, characterized by contrast retention in the lumen distal to the filter and no contrast filling proximal to the filter (**b**); after aspiration, partial restoration of IVC lumen was observed, accompanied by proximal contrast filling of the vena cava filter, although it was faint (**c**). Intraoperative angiography from the second thrombus aspiration procedure (**d-f**): the pre-aspiration angiography revealed extensive filling defects within IVC, with significant contrast stasis extending from the distal end to beneath the filter, and limited contrast reflux near the proximal end (**d**); post-aspiration angiography demonstrate a notable reduction in the extent of filling defects within IVC compared to the pre-aspiration state, additionally increased contrast enhancement is evident near the proximal end of the filter (**e**), and unobstructed right iliac vein blood flow is observed (**f**)

The patient was a 63-year-old male. He was involved in a vehicular accident, which resulted in a range of lower limb symptoms. The right lower limb exhibited numbness, redness, swelling, and a myriad of discomfort symptoms, including knee pain, stiffness, crepitus, ecchymosis, and restricted mobility. Diagnosed with a ‘right knee multiple ligament injury’, the patient’s condition was managed by the orthopaedic department, and a plan for anterior and posterior cruciate ligament reconstruction surgery was devised. The patient’s medical history was unremarkable. Upon admission, a lower limb venous ultrasound revealed thrombotic formations within the left popliteal vein, posterior tibial veins, and intramuscular veins of both calves. This situation met the criteria for prophylactic placement of an IVCF. Subsequently, the patient underwent an IVCF placement procedure utilizing the Denali™ filter (C. R. BARD, Inc.). Intraoperative observations indicated patent segments of the IVC (Fig. 1a). Postoperatively, the recommended regimen included low molecular weight heparin (LMWH) at a dosage of 4000 IU subcutaneously every 12 hours. However, the actual regimen was modified to a once-daily subcutaneous injection of LMWH. Soon afterwards, the scheduled surgical intervention could not be conducted within the initially projected timeframe as a result of objective factors, mainly including inadequate wound healing in the lower limbs. It is noteworthy that anticoagulation therapy had been discontinued 24 hours prior to the initially scheduled surgical time. Following the surgical delay, anticoagulant therapy was not promptly reinstated.

Three days after cessation of anticoagulant therapy, the patient experienced a sudden and severe bout of abdominal pain during the night, along with profuse sweating, palpitations, and a sense of unease accompanying the pain. The medical team promptly conducted a bedside assessment. The patient was alert, although he exhibited signs of mental distress. Shock symptoms were evident, including a blood pressure reading of 70/50 mmHg, a heart rate of 132 bpm, and a respiratory rate of 25 bpm. Despite receiving 3 L/min of supplementary oxygen, the patient maintained an SpO_2_ level of 100%. Abdominal tenderness was noted, while rebound tenderness was not pronounced. No significant abdominal muscle tension was observed, and the patient presented with bilateral pitting oedema in the lower limbs.

**Fig. 2:**
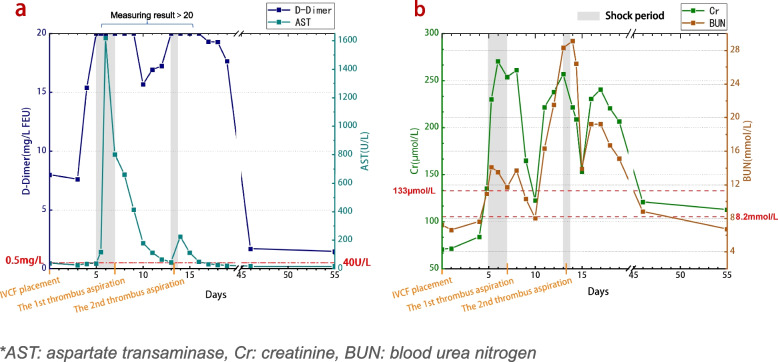
The changes in D-dimer and AST during the course of the illness (**a**). The changes in Cr and BUN during the same period (**b**). During the initial shock episode, the patient experienced severe impairment of liver and kidney functions. Following the first thrombus aspiration treatment, coupled with liver support and CRRT, liver function and kidney function exhibited gradual improvement within 3 days postoperatively. Subsequently, AST, Cr and BUN levels variably increased again prior to the occurrence of the second shock episode. After the second thrombus aspiration procedure, AST, Cr and BUN levels gradually decreased once more

**Fig. 3:**
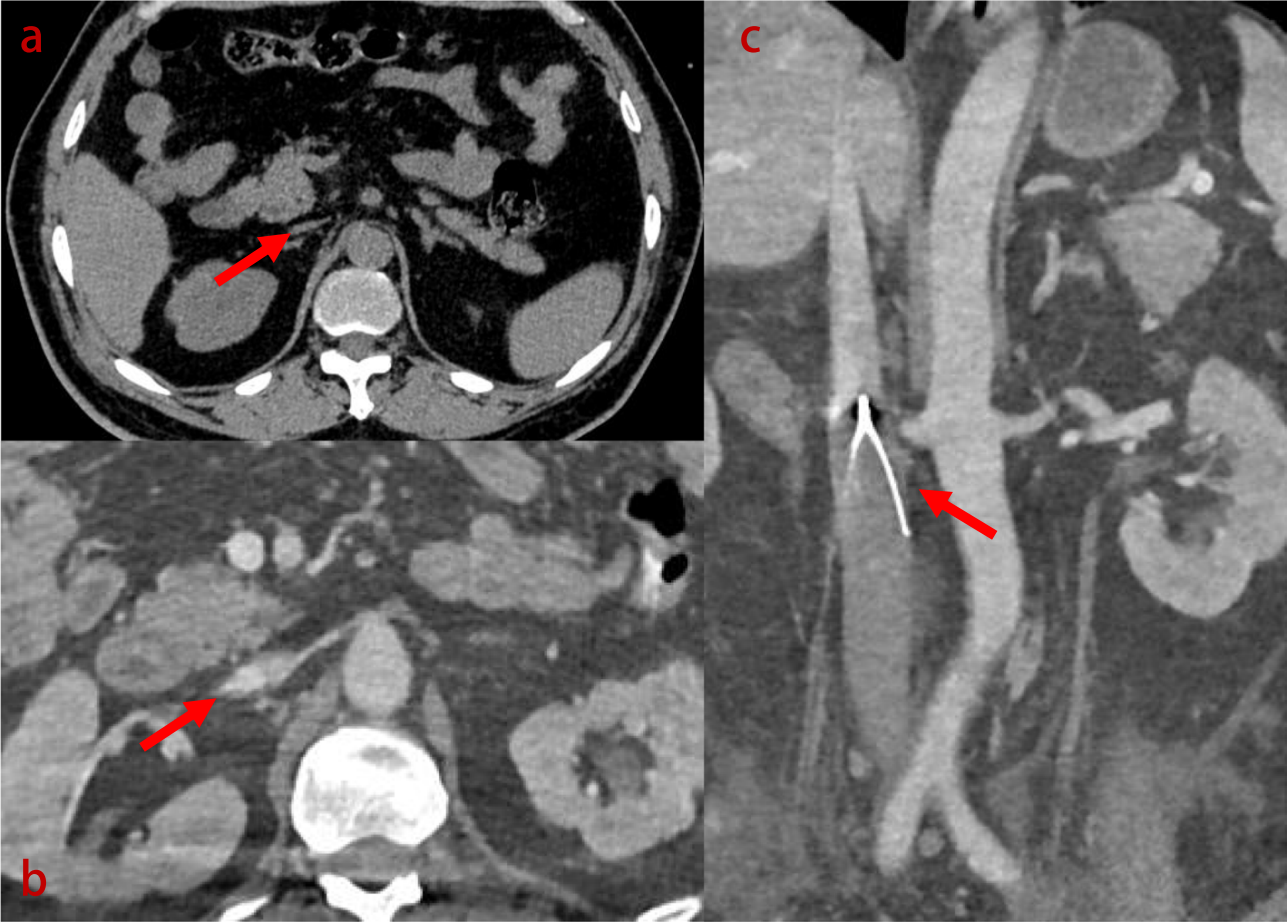
CT scan images, illustrating the collapse of the upper segment of IVC, with unchanged placement of the IVCF and no evident intra-abdominal haemorrhage (**a**). Further CTV images (**b** and **c**) revealed no contrast filling around and beneath the filter. Additionally, the vessels below the IVCF, including the bilateral common iliac veins and their distal branches, appeared engorged and enlarged. Contrast enhancement was poor in this area. Above the level of the IVCF, the vessel lumens appeared narrowed and flattened, with visible contrast enhancement. These findings collectively suggest thrombotic inferior vena cava occlusion below the IVCF

Despite aggressive fluid resuscitation and shock management, there was no significant improvement in blood pressure. Additionally, the patient remained anuric for a continuous 12-hour period. Following urinary catheterization, approximately 50 ml of haematuria was drained. At this point, the patient presented with marked oedema in both the lower limbs and the scrotum. Inflammatory markers exhibited a substantial elevation, and renal function began to show anomalies. D-dimer levels exceeded the upper limit of detection (Fig. 2a). Ultrasound examination revealed right cardiac contractility with an ejection fraction of 50%. The size of the cardiac chambers had notably decreased compared to the previous assessment (preoperative examination), suggesting changes possibly associated with decreased blood volume. No significant fluid collection was observed within the abdominal cavity, while peripheral oedema was evident. Chest and abdominal computed tomography scans revealed collapse of the upper segment of the IVC lumen (Fig. 3a).

Subsequent IVC contrast-enhanced computed tomography venography (CTV) revealed (Fig. 3b-c) that the vessel lumen exhibited narrowing and flattening above the IVCF, and, even with suboptimal contrast enhancement, both the bilateral common iliac veins and their distal branches below the filter appeared dilated and engorged. These findings are suggestive of a thrombotic occlusion involving the entire segment of the vasculature below the IVCF. There were also scattered filling defects detected in the main right pulmonary artery and several peripheral branches of both lungs, suggestive of PE. Correlating the examination results with the clinical presentation, we preliminarily deduced that extensive thrombus formation around the IVCF led to acute IVC obstruction. This resulted in a sudden decrease in cardiac return, subsequently precipitating obstructive shock.

During communication with the patient’s family, they requested temporary medical management. The patient received treatment including a crystalloid bolus infusion, vasopressor support, anti-infective and anticoagulation therapy (continuous infusion of unfractionated heparin at a total daily dose of 12,500 IU). Despite these interventions, shock symptoms remained uncorrected after 1 day. High-dose norepinephrine (150 μg/min) infusion was initiated to maintain blood pressure, which continued to fluctuate within the range of 90/45 mmHg to 95/55 mmHg. Concurrently, the patient remained anuric (Fig. 1), and severe impairment of liver and kidney function was evident (Fig. 2b). Meanwhile, the patient’s SpO_2_ remained challenging to maintain even with a flow rate of 15 L/min of oxygen, dropping to a nadir of 40%.

After further discussion with the patient’s family about indications and associated risks of surgical treatment, the family consented to the surgery. We promptly proceeded with the surgery. The initial angiography through the right popliteal vein confirmed the findings observed on the CT scan (Fig. 1b). Subsequently, we performed catheter-guided thrombus aspiration. We opted for the right popliteal vein as the access route. While advancing the guidewire through the popliteal vein to the segment below the filter within the IVC, we encountered substantial resistance. Subsequently, we switched to the Zelante thrombus aspiration catheter (8F) and performed repeated aspiration on the right femoral superficial vein, femoral vein, right common iliac vein, and IVC, with ANGIOJET™ Peripheral Thrombectomy System (Boston Scientific Corp.). Partial restoration of the catheter lumen was achieved, leading to improved blood flow compared to the previous state. The obstruction in the IVC was relieved to some extent (Fig. 1c). Due to approaching the upper limit in terms of both total aspiration volume and time, coupled with the patient’s coagulation dysfunction and widespread ecchymosis, the risk of bleeding associated with thrombolytic therapy was deemed high. As a result, we refrained from performing thrombus aspiration in the left lower limb veins and did not proceed with catheter-directed thrombolysis (CDT). Upon intraoperative observation of restored patency in the IVC, the patient’s hypotension was promptly alleviated. With a reduction in the norepinephrine infusion rate to 5 μg/min, the blood pressure stabilized at 120/70 mmHg. The patient also began to pass clear, pale yellow urine. Postoperatively, urinary output increased to 2250 ml in 24 hours (Fig. 1). Swelling in the lower limbs notably diminished compared to their preoperative status.

Following the surgery, the patient remained in the ICU and received continuous anticoagulation therapy (infusion of argatroban at a daily total of approximately 40–60 mg, with an activated partial thromboplastin time maintained at 50–60 seconds), vasopressor therapy, liver support, and continuous renal replacement therapy (CRRT). The patient’s blood pressure stabilized, and daily urine output averaged approximately 4000–6000 ml. During this period, thrombophilia screening, including tests for protein C, protein S, antithrombin III (AT III), and autoimmune-related markers, yielded negative results. Upon achieving a relatively stable condition, the patient was transferred from the ICU to a general ward for continued treatment. Unfortunately, soon after the transfer, the patient experienced another episode of decreased blood pressure and anuria (Fig. 1). An urgent bedside ultrasound examination was conducted, revealing a collapse of the upper segment of the IVC. This finding led to the suspicion of extensive thrombosis around the IVCF, accompanied by shock. Subsequently, an emergency surgical intervention was performed. Using bilateral popliteal vein access, venography of both lower limbs and the IVC was conducted, followed by thrombus aspiration (Fig. 1d-f). After repeated thrombus aspiration, the patient’s blood pressure and urine output rapidly recovered once again (Fig. 1).

After repeated thrombus aspiration treatment, continuous anticoagulation therapy (continuous infusion of argatroban) and other symptomatic and supportive treatments were given. Once the patient’s condition stabilized, oral anticoagulant therapy with apixaban (2.5 mg po bid) was initiated. Approximately 1 month later, the patient’s overall condition and liver and kidney function gradually recovered to normal (Fig. 2b). Subsequently, the patient was discharged in a stable condition.

**Fig. 4:**
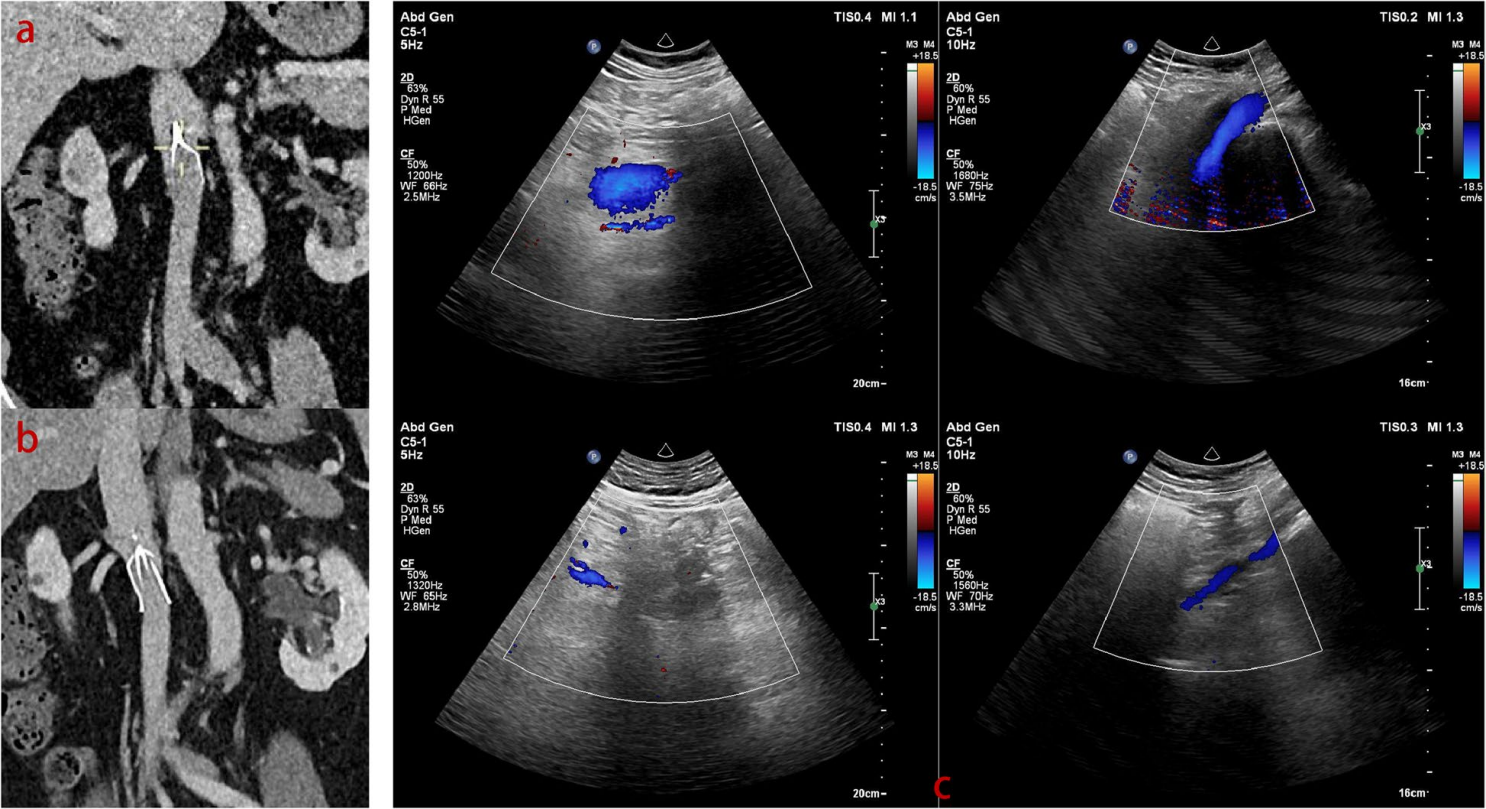
Postoperative CTV images obtained 1 month after the procedure (**a**). Below the IVCF, both iliac veins and their distal branches exhibit increased calibre and fullness, with suboptimal contrast enhancement. Suspected scattered thrombi are visible throughout the entire segment of vessels below the IVCF, and the previously observed low-density areas have diminished. CTV images obtained 3 months after the procedure, depicting unobstructed contrast enhancement within the IVC and its branches, with no evident filling defects (**b**). Ultrasound images of IVC and iliac veins obtained 12 months postoperatively (**c**). The IVC displays robust blood flow, with normal flow direction. Flow is unobstructed in the common and external iliac veins. In the right calf, partial thrombosis of the intermuscular veins accompanied by venous calcification is observed, while the remaining deep and superficial veins exhibit unobstructed return flow

One month postoperatively, although follow-up examination revealed scattered thrombi below the filter, there were fewer than found in the previous examination (Fig. 4a). Approximately 50 days after the second thrombus aspiration surgery, the patient successfully underwent knee joint surgery. Follow-up evaluations at 3 and 12 months showed favourable results, with the IVC and the inserted filter demonstrating good blood flow and unobstructed bilateral iliac vein return (Fig. 4b-c). Thrombophilia and haemostasis gene *panel* testing revealed no detected mutations in relevant genes.

## Discussion

DVT is one of the most common and serious complications in patients with traumatic fractures [1]. According to Virchow’s triad, traumatic fracture patients are susceptible to DVT due to a combination of factors, including vascular wall injury, sluggish blood flow (due to immobilization), and a hypercoagulable state (resulting from activation of the coagulation system after trauma). Consequently, the incidence of DVT is relatively common, occurring at a rate of 1.16%. Furthermore, the likelihood of lower limb DVT embolization inducing the development of PE is approximately 0.93%, and the mortality rate associated with PE varies between 0.38 and 13.8%, making it a significant contributor to perioperative mortality [2]. Placement of an IVCF is crucial for preventing PE [3], and it has been demonstrated to reduce the occurrence rate of PE from 60 to 70% to 0.9–5.0% [4]. An analysis of the United States National Trauma Database revealed a negative correlation between prophylactic IVCF insertion in orthopaedic trauma patients at higher risk for VTE and overall in-hospital mortality rates [5].

However, everything has its pros and cons. While an IVCF effectively intercepts the detachment of DVT and prevents the development of severe complications associated with the lung, thereby reducing the occurrence of PE [6], the potential complications of IVCF application cannot be overlooked. With the increasing use of IVCF, both early and late complications have been observed. The former includes puncture site complications, improper positioning, delivery system complications, tilting, and incomplete deployment; the latter includes IVC-related thrombosis, filter fracture, vascular perforation, and vascular-enteric fistula [7, 8]. Research has found that IVCT is the most common complication of filter implantation [9], and the presence of the filter increases the risk of lower extremity DVT, particularly when anticoagulation therapy is not administered [10]. A meta-analysis conducted by Angel et al. [11] revealed an occurrence rate of 4.1% for IVC thrombosis or occlusion after IVCF insertion, with symptomatic cases accounting for 0.8% [11]. IVCT is one of the causes of inferior vena cava syndrome (IVCS) [10]. When there is significant obstruction in the IVC, the venous pressure downstream, including pelvic veins and lower extremity veins, increases. This can result in the formation of ascites, genital oedema, and lower extremity swelling. In chronic cases, the development of acute lower extremity oedema may not occur due to the establishment of collateral circulation, while in acute cases, it can cause bilateral lower extremity oedema, cyanosis of the thighs, or even phlegmasia alba dolens, endangering the affected limb. IVCS symptoms can vary greatly. Patients may be asymptomatic or have mild symptoms. Acute or subacute IVCT patients may complain of abdominal pain, back pain, and/or pelvic pain radiating to the groin [10, 12]. Currently, cases of IVCT leading to shock are extremely rare.

In this case, the patient developed left popliteal vein and posterior tibial vein thrombosis after sustaining traumatic bone fractures. 5 days after undergoing IVCF placement, the patient suddenly experienced abdominal pain accompanied by haemodynamic instability, tachycardia and anuria. Subsequently, the patient gradually developed tachypnea and altered consciousness. While actively managing shock and providing supportive care, we conducted relevant laboratory and imaging tests to determine the type and underlying cause of the shock.

The progression from abdominal pain to a state of shock was sudden. While inflammatory markers such as white blood cell count, C-reactive protein and procalcitonin were elevated, the patient did not exhibit stress-induced hypertension, fever, rebound tenderness, or abdominal muscle tension, and blood microbial cultures did not yield positive results. Despite diagnostic treatment, such as adequate fluid resuscitation, vasopressors, and empirical use of broad-spectrum antibiotics for suspected infection, the patient’s blood pressure did not improve significantly. As a result, the possibility of infectious or hypovolemic shock appeared unlikely at this stage. Further investigations, including comprehensive cardiac enzyme analysis, echocardiography, and electrocardiography, failed to yield meaningful positive results, making cardiogenic shock less likely. The patient’s overall condition continued to deteriorate, and multiple organ dysfunction syndrome (MODS) emerged. Glutamic pyruvic transaminase and AST levels were elevated significantly, ranging 6–40 times higher than normal, blood Cr exceeded 176.8 μmol/L with either anuria or oliguria, 24-hour urine output was limited to 50–350 ml, lactate dehydrogenase levels were markedly elevated at 577 U/L, prothrombin time and thrombin time showed abnormal results, white blood cell count surpassed 12.0 × 10^9^/L, and there was altered consciousness.

Subsequently, the patient developed significant swelling in both the lower limbs and the scrotum, providing a new clue: the possibility of venous obstruction. A follow-up CT scan confirmed our suspicions, revealing collapse of the upper segment of the IVC. Considering the patient’s history of IVCF placement, suboptimal anticoagulation treatment postoperatively, and the notable elevation in D-dimer levels, these factors led us to focus on the possibility of inferior vena cava obstruction.

We conducted a further CTV examination, and the results showed that the vessels below the IVCF, including the bilateral common iliac veins and their distal branches, appeared engorged and enlarged. Contrast enhancement was poor in this area. Above the level of the IVCF, the vessel lumens appeared narrowed and flattened, with visible contrast enhancement. Taking into consideration these imaging findings, along with the previously noted reduction in cardiac chamber size suggestive of reduced preload and the suboptimal anticoagulation therapy following the initial IVCF placement (which was only briefly administered and at an insufficient dose), we can deduce that the acute occlusion of the iliac venous system below the IVCF was due to thrombotic formation. This occlusion led to a sudden decrease in the cardiac preload and obstructed venous return from the lower limbs. The latter directly resulted in symptoms such as lower limb and perineal swelling, while the former was the main factor causing obstructive shock, which was responsible for the patient’s hypotension and anuria.

Obstructive shock is a condition characterized by acute blood flow obstruction in the central vessels of either the systemic or pulmonary circulation, leading to shock. Its clinical manifestations vary depending on the severity and abruptness of vessel damage, including altered consciousness, hypotension, tachycardia, oliguria, and tachypnea. The occurrence of obstructive shock is extremely rare, accounting for less than 1% of shock cases [13]. Mechanical factors associated with obstructive shock can reduce blood flow in major vessels or within and outside the vascular lumen, resulting in a significant decrease in cardiac output and systemic oxygen supply. Consequently, rapid and substantial reductions in cardiac output and blood pressure ensue, leading to a shock state of tissue hypoxia affecting all organ systems. Immediate treatment is crucial to relieve the obstruction causing obstructive shock. The management of obstructive shock secondary to inferior vena cava obstruction depends on the chronicity of thrombosis and other associated complications. In most cases, patients can be successfully relieved through anticoagulation therapy, catheter-guided thrombolysis, or thrombectomy, as reported in the literature [6, 14, 15]. In the present case, we performed catheter-directed thrombus aspiration to relieve the IVC obstruction, leading to an immediate recovery of blood pressure and subsequent restoration of urine output (Fig. 1). Furthermore, the postoperative reduction in lower limb swelling and improvement in tissue tension compared to preoperative conditions highlight the significant therapeutic effect.

We conducted a retrospective analysis of previously reported cases of obstructive shock secondary to IVCT. Using the keywords “inferior vena cava thrombosis” and “obstructive shock,” we searched the PubMed database and identified a total of 3 relevant articles (Table 1). The three cases of obstructive shock following IVCT shared common characteristics: a history of previous placement of IVCF, typical clinical manifestations such as tachycardia, hypotension, altered consciousness, and peripheral signs, extensive thrombosis causing near-complete occlusion of the iliac venous system, initial treatment including antimicrobial therapy, fluid resuscitation, and vasopressors, targeted intervention for obstructive shock following diagnosis, and rapid resolution of shock symptoms after relief of the obstruction [6, 13, 16]. Among these cases, one report by E.K. Pearce et al. shares similarities with the present case. The patient was scheduled for cervical laminoplasty and had discontinued anticoagulation treatment and received an IVCF placement 1 day prior to the procedure. Subsequently, acute iliac venous system thrombosis occurred [13]. Thrombus aspiration was employed in three cases [6, 13, 16], and catheter-directed thrombolysis (CDT) was used in one case [13].

**Table 1:** Case reports of shock due to inferior vena cava thrombosis

Year	Author	IVCF placement	History/ Cause	Cardinal symptom						Thrombus site	Intervention	Short term outcome	Long term outcome
				Hypotension	Tachycardia	Tachypnea	Peripheral oedema	Dysuria	Confusion				
2016	Chen YL. et al. [18]	No VA-ECMO* catheter implanted	Inadequate anticoagulation	Unspecified						From IVC to atrium	Pharmacologic anticoagulation	Thrombus disappeared after 23 days of heparin anticoagulation	Good
2018	Mohammed M. et al. [6]	Yes	Road traffic accident IVCF placement without retrieval 15 years prior	Yes	Yes	Yes	Yes	Yes	Yes	From bilateral common femoral veins to the distal IVC	Mechanical thrombectomy	Shock was resolved	Good
2021	Pearce EK. et al. [13]	Yes	Inadequate anticoagulation	Yes	Yes	No	Yes	No	Yes	From bilaterally femoral veins to the IVCF	CDT Suction thrombectomy	Shock was resolved and pressor support was discontinued	Good
2021	Siddiqui S. et al. [17]	Yes	Road traffic accident IVCF placement without retrieval 15 years prior Thrombophilia	Yes	Yes	No	No	No	Yes	IVCF was occluded	IVCF retrieved Revascularization	Blood pressure and symptoms were improved	Undspecified
2023	Lane OF. et al. [16]	Yes	IVCF placement without retrieval 12 years prior	Yes	Yes	Yes	No	No	No	From bilateral common iliac veins to the IVCF	Thrombectomy	Blood pressure immediately increased with rapid downtrending of vasopressor requirement	Good

*VA-ECMO: venoarterial extracorporeal membrane oxygenation

Expanding the search scope and changing the keywords to “inferior vena cava thrombosis” and “shock” yielded 2 additional relevant articles related to the topic (Table 1). In one case, the patient had a significant history of retaining an IVCF for 12 years and had antiphospholipid syndrome. There were limited follow-up records for the patient, and compliance with anticoagulation treatment was questionable. Upon examination, complete thrombosis of the IVCF was observed, with extensive collateral circulation formed in the pelvis and lumbar vertebrae (occluded IVCF, pelvic and lumbar collateral vessels were extensively formed) [17]. In another case, the intensity of the patient’s anticoagulation therapy was reduced after ECMO to mitigate the risk of cerebral haemorrhage. The ECMO cannulas were inserted via the right femoral vein and left femoral artery, with the distal end positioned at the level of the right atrium. Subsequently, the patient developed floating thrombi from the IVC to the right atrium. In both cases, thrombus formation occurred in different segments of the IVC due to the presence of intracavity foreign bodies and inadequate anticoagulation treatment, resulting in various degrees of acute and chronic manifestations [18]. These two patients experienced symptom improvement in the short term following vascular reconstruction and medical treatment, with favourable long-term recovery.

An early randomized prospective study [19] found that in patients with proximal DVT, those who received IVCF along with anticoagulation treatment had a higher DVT recurrence rate than those who received anticoagulation treatment alone (permanent filters were used in this study) [19]. The increased risk was attributed to the combination of venous haemodynamic changes and blood stasis caused by the filter, along with potential coagulation dysfunction in the patients. A retrospective study evaluating the incidence and risk factors for IVCT after IVCF placement indicated that discontinuation of anticoagulation therapy after filter placement was a risk factor for IVCT occurrence [12]. In this case, after the patient’s condition stabilized in the ICU and he was transferred to a regular ward, he again experienced symptoms such as oliguria and decreased blood pressure. Imaging and contrast examination confirmed extensive thrombus formation in the IVC once again. It might be challenging to definitively establish a direct relationship between the two occurrences of IVC thrombosis and factors such as the filter, anticoagulation treatment intensity, or whether massive blood clots fell off. We analysed the reasons for recurrent IVCT and concluded that it could be attributed to the interruption of anticoagulation treatment during the transition between different wards, thereby resulting in inadequate and nonstandardized anticoagulation therapy. Alternatively, during patient transportation, manipulation of the lower limbs might have dislodged and caused thrombi to occlude the IVC. There is still a lack of specific research on the relationship between complications arising after IVCF placement and the corresponding treatment requirements. Relevant literature suggests that in cases of IVCS associated with thrombosis, long-term anticoagulation treatment might be the preferred choice, particularly in the absence of identifiable triggering mechanisms [10].

In cases involving intravascular implants such as IVCF, we believe it is crucial to ensure adequate anticoagulation therapy. Health care professionals should consider the patient’s drug responsiveness and the mechanisms of action of different anticoagulant medications to select the appropriate treatment regimen. Additionally, clinicians should enhance their ability to recognize IVCS and obstructive shock. In the event of obstruction, prompt intervention should be based on the underlying cause. For cases of obstructive shock resulting from IVCT, various treatment modalities can be considered, including thrombus aspiration, CDT, vascular reconstruction, and medication therapy. Based on our center’s experience, mechanical thrombus aspiration surgery has shown favourable outcomes for acute IVC thrombotic obstruction and could be a valuable approach to consider.

**Fig. 5:**
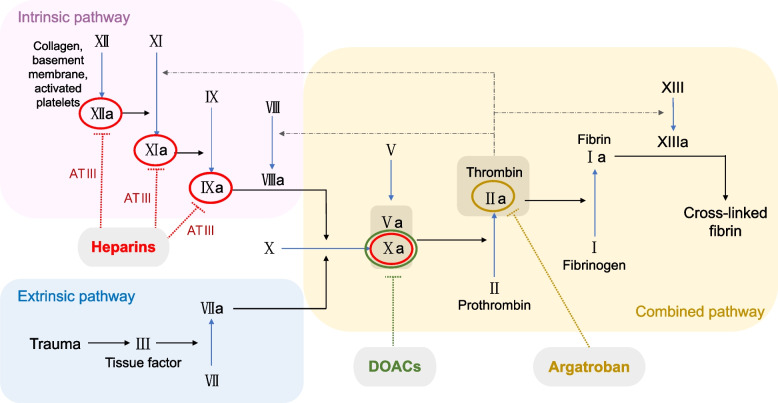
Coagulation mechanism and targets of common medications

Additionally, throughout the treatment process, we made some adjustments to the use of anticoagulant medications. Initially, during the detection of lower limb DVT and the placement of IVCF, we followed the classic approach of using LMWH 4000 U subcutaneously every 12 hours, which is a commonly recommended prophylactic regimen for VTE by organizations such as the Eastern Association for the Surgery of Trauma (EAST) and the American College of Chest Physicians (ACCP). They recommend LMWH for VTE prophylaxis in general trauma patients [20]. LMWH has a relatively short half-life, usually requiring discontinuation 24 to 12 hours before surgical procedures. On the other hand, while direct oral anticoagulants (DOACs) have advantages such as stronger anticoagulant effects than LMWH and warfarin, reduced bleeding risk, and convenience of use, they typically require a 3-day period of discontinuation before surgery. This prolonged period without anticoagulation intervention may increase the risk of VTE. Later, when IVCT occurred and we observed ongoing thrombus formation during LMWH use, we considered that it would not be suitable for patients with renal insufficiency and instead opted for continuous infusion of unfractionated heparin to achieve more stable blood drug concentrations. Heparin drugs directly target antithrombin III (AT III), significantly enhancing its activity. AT III then binds to factors such as XIIa, XIa, IXa, and Xa, inhibiting their activity and exerting anticoagulant effects (Fig. 5). However, after 24 hours of conventional heparin treatment, there was no significant improvement in the patient’s symptoms. We suspected that the patient might not be sensitive to heparin-class anticoagulant drugs. Therefore, after the first thrombus aspiration procedure, we switched to an argatroban infusion. Argatroban is a direct inhibitor of factor IIa (thrombin), which inhibits fibrin formation directly without depending on AT III (Fig. 5). As the patient’s condition gradually stabilized, for oral medication, we also opted for DOACs that directly inhibit factor Xa and do not rely on AT III. Considering the patient’s severe renal impairment, among the commonly used DOACs, including rivaroxaban, apixaban, and edoxaban, we finally chose apixaban, which has minimal renal metabolism and is less affected by renal dysfunction. The patient was given half of the standard anticoagulant dose (2.5 mg po bid).

## Conclusion

IVCF holds significant importance because it plays a crucial role in preventing the embolization of lower limb DVT to vital organs, especially the lungs, effectively reducing the incidence of PE. However, every coin has two sides. While filters fulfil their mission, the complications they bring should not be overlooked. Clinical practitioners should enhance their understanding of the prevention, recognition, and corresponding treatment measures for filter-related complications and remain vigilant in preventing severe complications. Post-IVCF implantation, meticulous postoperative management is essential. Ensuring standardized and adequate anticoagulation therapy is crucial, and the choice of anticoagulant drugs should be tailored to the patient’s specific conditions. If a patient presents with symptoms such as hypotension, tachycardia, and lower limb and scrotal oedema, immediate consideration should be given to the possibility of obstructive shock and clarification of its underlying cause. In the event of acute inferior vena cava thrombotic obstruction, mechanical thrombus aspiration surgery demonstrates significant efficacy and a favourable prognosis. Alternatively, different treatment modalities, such as CDT, vascular reconstruction, and medical therapy, can be selected based on the individual patient’s circumstances.

## Data Availability

Not applicable.
